# A case of successfully resected metachronous gastric and gallbladder metastases from pancreatic body cancer

**DOI:** 10.1186/s40792-019-0581-1

**Published:** 2019-02-13

**Authors:** Makoto Takahashi, Hideyuki Yoshitomi, Atsushi Kato, Katsunori Furukawa, Tsukasa Takayashiki, Satoshi Kuboki, Shigetsugu Takano, Kensuke Sugiura, Keishi Kawasaki, Masaru Miyazaki, Masayuki Ohtsuka

**Affiliations:** 1 Department of General Surgery, Chiba University, Graduate School of Medicine, 1-8-1 Inohana, Chuo-ku, Chiba-shi, Chiba, 260-8670 Japan; 2 Digestive Diseases Center, International University of Health and Welfare, Mita Hospital, Tokyo, Japan

**Keywords:** Pancreatic cancer, Metachronous gastric metastasis, Metachronous gallbladder metastasis, Surgery

## Abstract

**Background:**

Pancreatic ductal adenocarcinoma (PDAC) readily metastasizes to the lymph nodes, liver, lung, and peritoneum; however, gastric and gallbladder metastases are rare. We report a case of metachronous gastric and gallbladder metastases from PDAC.

**Case presentation:**

The patient is a 71-year-old man who underwent distal pancreatectomy for PDAC. Seventeen months after the surgery, a 30-mm nodule was detected at the lesser curvature of the stomach, which was diagnosed as recurrence of PDAC in the lymph nodes. He then received gemcitabine and S-1 combination chemotherapy for 6 months. Because tumor size remained approximately the same and tumor marker levels decreased, total gastrectomy and cholecystectomy were performed. Pathological examination showed well-differentiated tubular adenocarcinoma in the subserosa and muscularis propria of the stomach and gallbladder. The patient remains alive at 41 months after the second surgery with liver metastasis.

**Conclusion:**

We reported a rare case of metachronous gastric and gallbladder metastases from pancreatic body cancer.

## Background

Prognosis in patients with pancreatic ductal adenocarcinoma (PDAC) remains dismal, with a 5-year survival rate below 10% [1, 2]. The lymph nodes, liver, lung, and peritoneum are the predominant sites of metastasis; however, gastric and gallbladder metastases are very rare. We describe a case of metachronous gastric and gallbladder metastases from pancreatic body cancer.

## Case presentation

**Fig. 1:**
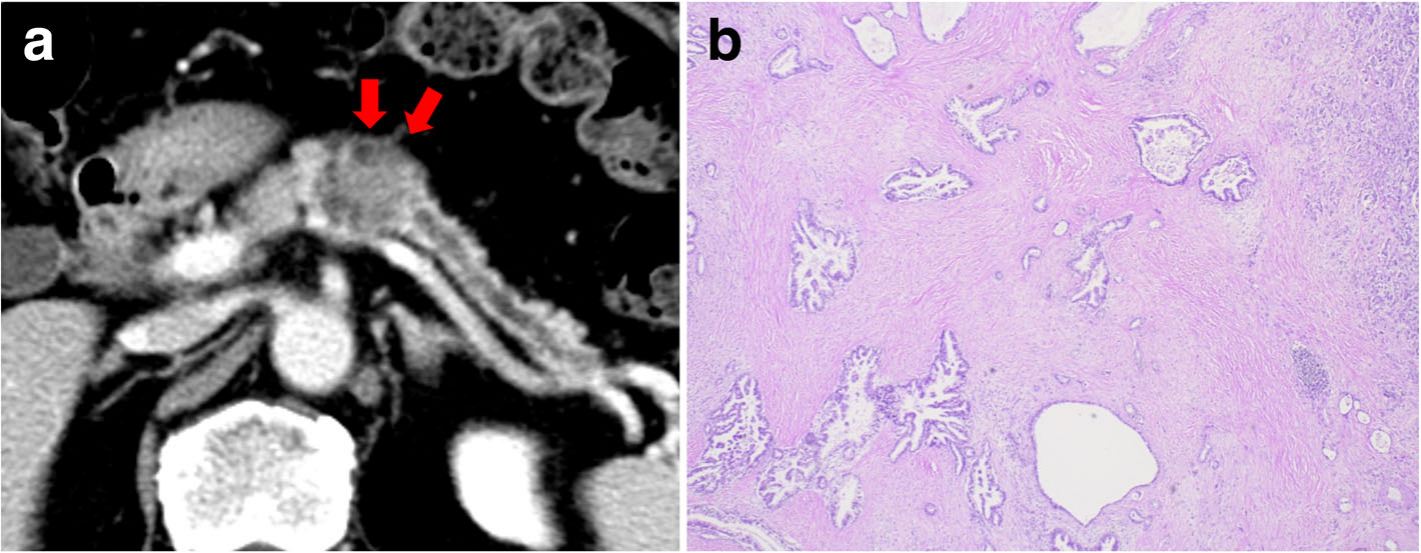
Primary operation for pancreatic body cancer. **a** Multi-detector row computed tomography (MDCT) showed pancreatic body cancer before the first surgery (arrows). **b** Pathological findings of the primary tumor were as follows: invasive ductal carcinoma, well-differentiated type, and (T3, N0, M0) stage IIa

**Fig. 2:**
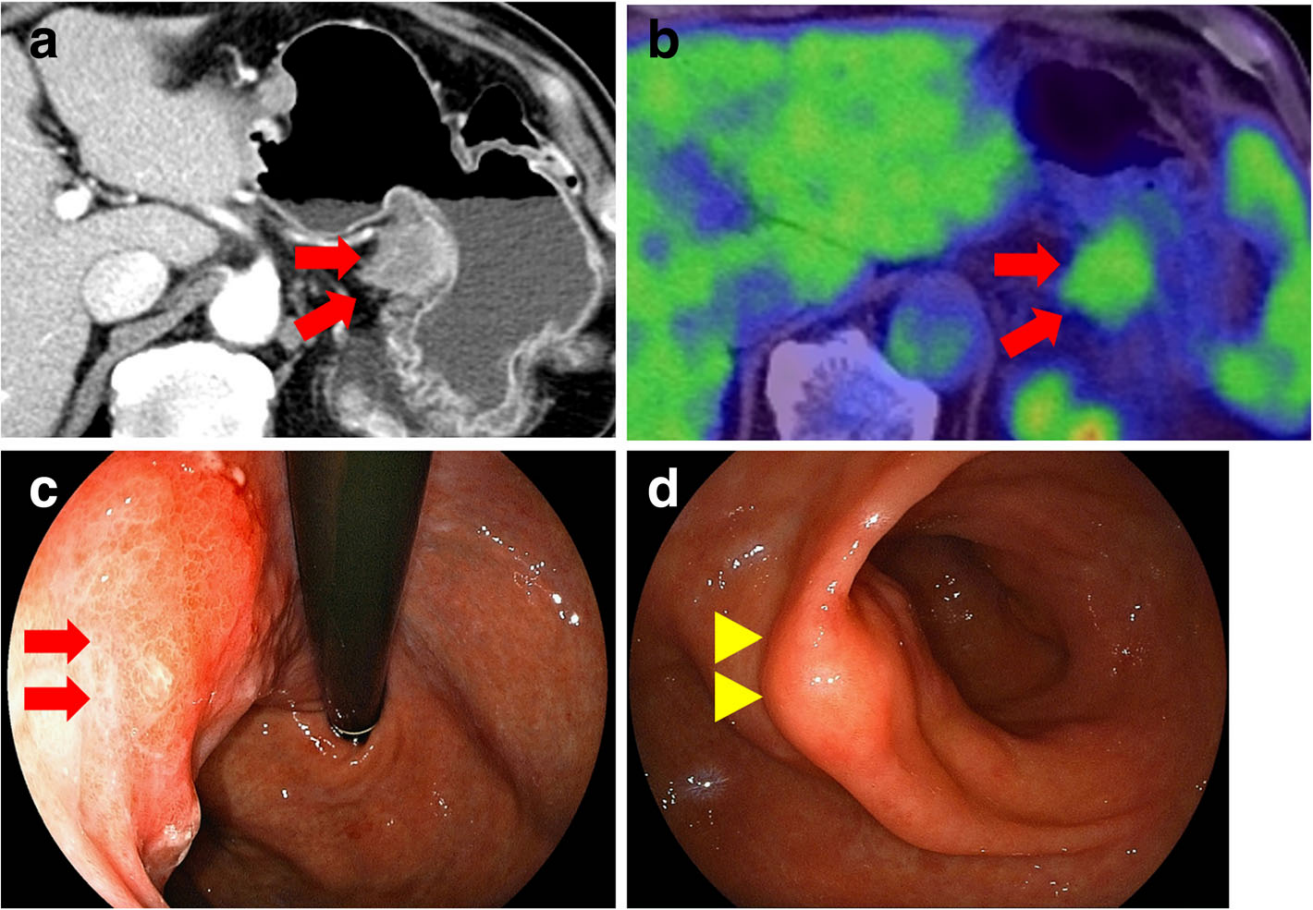
Imaging diagnosis of recurrence. **a** MDCT showed a 30-mm nodule at the lesser curvature of the stomach. **b** Positron emission tomography (PET)/CT showed fluorodeoxyglucose (FDG) uptake in the nodule; SUVmax was 3.5. **c** Upper gastrointestinal endoscopy revealed mucosal irregularity in the posterior wall of the lesser curvature of the gastric body (arrow). **d** Upper gastrointestinal endoscopy revealed submucosal tumor in the anterior wall of the gastric antrum (arrowhead)

A 71-year-old man with a history of atrial fibrillation, acute appendicitis, and early esophageal cancer treated with endoscopic submucosal dissection underwent distal pancreatectomy with splenectomy for treatment of pancreatic body cancer (Fig. 1a). Pathological examination showed well-differentiated tubular adenocarcinoma (pT3, pN0, pM0). The Union for International Cancer Control (UICC) stage was established as IIa (Fig. 1b). After surgery, the patient received adjuvant chemotherapy of S-1 for 6 months. Multi-detector row computed tomography (MDCT) showed a 30-mm nodule at the lesser curvature of the stomach 17 months after pancreatectomy during postoperative surveillance (Fig. 2a). Positron emission tomography (PET)/CT showed fluorodeoxyglucose (FDG) uptake in the nodule; maximum standardized uptake value uptake (SUVmax) was 3.5 (Fig. 2b). Upper gastrointestinal endoscopy revealed mucosal irregularity in the posterior wall of the lesser curvature of the gastric body (Fig. 2c) and submucosal tumor in the anterior wall of the stomach antrum (Fig. 2d). Endoscopic ultrasound (EUS) showed the hypoechoic submucosal tumor, which was diagnosed as adenocarcinoma by fine-needle aspiration (FNA) cytology.

Based on these findings, we diagnosed lymph node metastasis of PDAC invading the gastric wall, and the patient was treated with gemcitabine and S-1 combination (GS) therapy for 6 months. Considering the patient’s age and prior pancreatic resection, we selected GS therapy due to its relatively high response rate and manageable complication rate found in a previous phase III study for unresectable pancreatic cancer (GEST study) [3]. Because the tumor size did not change over 6 months and CA 19-9 decreased from 54 to 28 U/mL, we decided to perform total gastrectomy and prophylactic cholecystectomy for tumor resection.

**Fig. 3:**
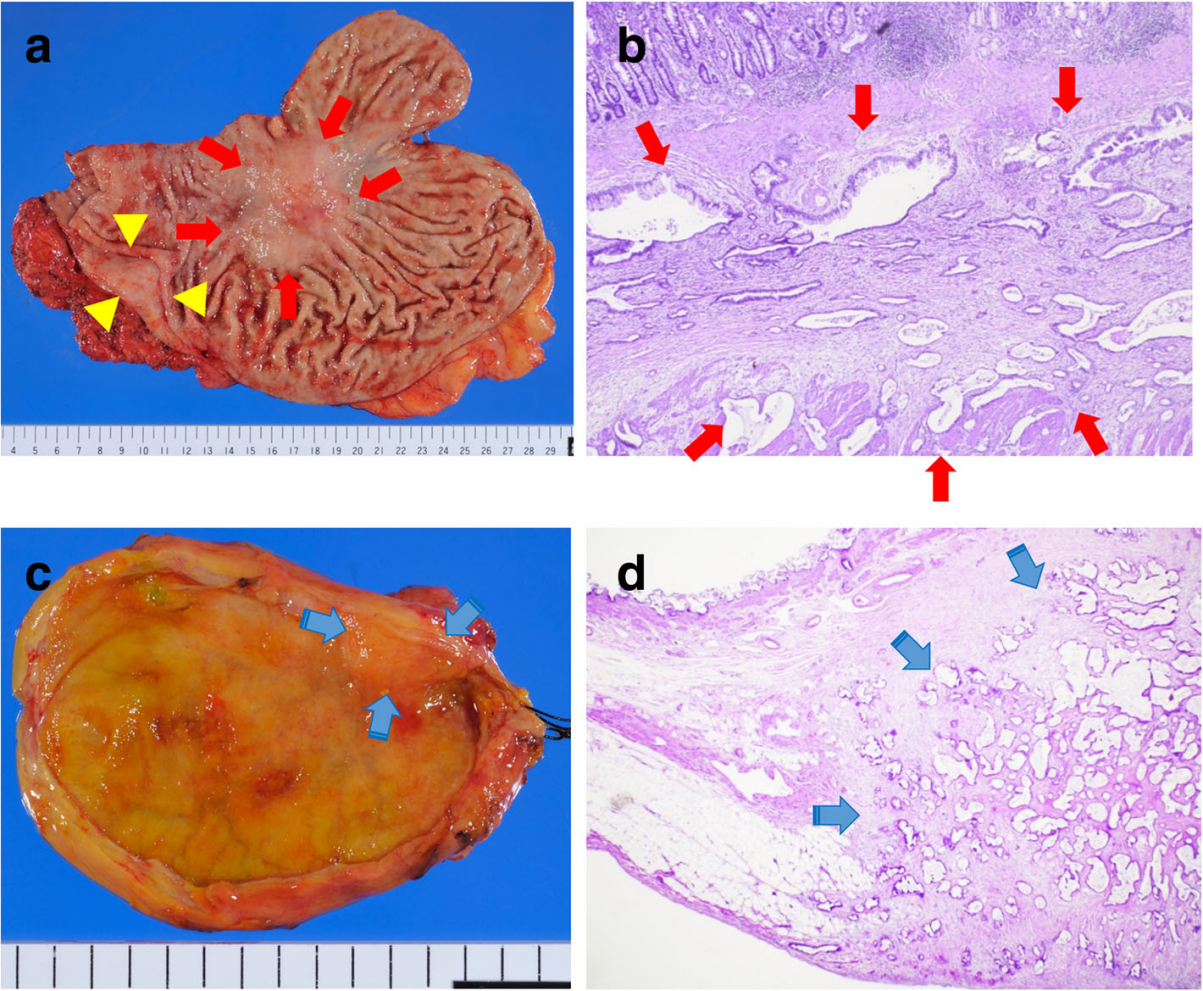
Histological findings of metastases. **a** Macroscopic findings in the stomach. There were two lesions in the stomach wall (arrow: lesser curvature (25 × 20 mm), arrowhead: anterior wall of antrum (15 × 13 mm). **b** Pathological examination showed well-differentiated tubular adenocarcinoma in the subserosa and muscularis propria of the stomach; the tumor was not exposed to the mucosa and serosa (arrow), and there was no involvement of lymph nodes. **c** Macroscopic finding in the gallbladder. A 13 × 5-mm submucosal tumor was found in the neck of the gallbladder (arrow). **d** Well-differentiated tubular adenocarcinoma located in the subserosa of the gallbladder (arrow)

Macroscopically, two nodules were located in the stomach, with one in the wall of the lesser curvature and the other in the antrum anterior wall (Fig. 3a). Pathological examination showed that these were well-differentiated tubular adenocarcinomas in the subserosa and muscularis propria of the stomach. The tumor was not exposed to the mucosa and serosa, and there was no lymph node involvement (Fig. 3b).

Surprisingly, there was a tumor in the neck of gallbladder; pathological examination revealed this was a well-differentiated tubular adenocarcinoma in the subserosa (Fig. 3c, d).

As the pathological findings of these tumors were similar to the lesion initially resected by distal pancreatectomy, we diagnosed metachronous gastric and gallbladder metastases from PDAC.

The patient’s postoperative course was uneventful, and the patient was discharged on postoperative day 16. Because of prior S-1 adjuvant therapy after pancreatectomy and GS therapy before gastrectomy/cholecystectomy, we elected to defer further chemotherapy. A liver metastasis was discovered 7 months after the second operation. He was then treated with GS therapy followed by gemcitabine and albumin-bound paclitaxel combination therapy and modified FOLFIRINOX therapy. At 41 months after the second surgery, the patient remained alive.

## Discussion

Previous studies have noted that the predominant sites of metastasis for PDAC are the lymph node, liver, lung, and peritoneum [4, 5]. We described a rare case of metachronous gastric and gallbladder metastases from pancreatic body cancer. Gastric metastasis of PDAC is rare. To our knowledge, only three cases have been reported in the English literature [6–8]. Oda et al. performed autopsies on 209 patients with PDAC and reported that gastric metastasis was found in only two cases [9].

Generally speaking, there are five metastatic pathways to the stomach: (1) direct invasion, (2) intraoperative seeding, (3) hematogenous metastasis, (4) lymphatic metastasis, and (5) intraluminal or intramural dissemination [10]. In our case, the primary pancreatic tumor was not anatomically near the stomach, and resection with tumor-free margins was performed. In addition, the two metastatic lesions in the subserosa and muscularis propria of the stomach did not expose to the mucosa or serosa. Considering these facts, we thought that gastric metastasis occurred through the hematogenous pathway.

Gallbladder metastasis of PDAC is also rare. This appears to be the first report of resected gallbladder metastasis from PDAC in the English literature. An autopsy study by Kishi et al. demonstrated a 7.4% incidence of gallbladder metastasis among patients with PDAC [11]. Yoon et al. surveyed 417 patients with pathologically confirmed gallbladder malignancies; 20 (4.8%) were metastatic (stomach, *n* = 8; colorectum, *n* = 3; liver, kidney, and skin, *n* = 2 each; and others, *n* = 3) [12]. In our case, as the tumor was subserosal and did not expose to the mucosa, hematogeneous metastasis was most likely, as with the gastric metastases. Although Yoon et al. also reported that the prognosis of metastatic gallbladder carcinoma was poor, with 8.7 months of median overall survival [12], our case resulted in long-term survival after surgical resection.

There was a possibility that cancer cell seeding by FNA was the cause of gastric metastases. However, we think it unlikely because one of the metastases was located in the antrum, far from the aspiration site, and we also found a gallbladder metastasis which could not have been related to FNA.

Surgical resection for disease relapse of PDAC remains debatable due to lack of evidence of survival benefit. Recently, several studies showed survival benefit of surgical resection for PDAC recurrence. Miyazaki et al. showed a prolonged survival by repeat pancreatomy for isolated local recurrence [13]. Thomas et al. highlighted a single tumor as a key factor benefiting most from re-resection for lung metastasis from PDAC [14].

In the case presented here, we first diagnosed solitary lymph node recurrence of PDAC after pancreatectomy by CT imaging and performed surgical resection after chemotherapy. Unintentionally, two gastric metastases and one gallbladder metastasis were resected with tumor-free margins. This patient’s survival of more than 3 years suggests the benefit of surgical intervention in combination with systemic chemotherapy. However, specific selection criteria for resection for multiple metastases remain unclear.

## Conclusions

We reported a case of surgically resected metachronous gastric and gallbladder metastases from pancreatic body cancer. Further studies of surgical resection of PDAC recurrence are needed.
